# Supplementary material for: Co-assessment of costs and environmental impacts for off-grid direct air carbon capture and storage systems
Source: Commun Eng. 2024 Jan 16;3:14. doi: 10.1038/s44172-023-00152-6 (PMC10956006; doi:10.1038/s44172-023-00152-6)
Supplement: Supplementary file 2 — Supplementary Information [file 44172_2023_152_MOESM2_ESM.pdf]

Supplementary information

Co-assessment of costs and environmental impacts for off-grid direct air carbon capture and storage systems

Moritz Gutsch[1,2,*] , Jens Leker[1,2]

[1] Helmholtz Institute Münster, IEK-12, Forschungszentrum Jülich GmbH, Corrensstraße 46, Münster 48149, Germany

[2] Institute of Business Administration at the Department of Chemistry and Pharmacy, University of Münster, Leonardo Campus 1, Münster 48148, Germany

[*] Corresponding author. E-mail address: moritz.gutsch@uni-muenster.de

Content

1. Supplementary Note 1: Literature overview
2. Supplementary Note 2: Input data for costs and environmental impacts

1. Literature overview

1.1. Life cycle assessments (Global warming potential)

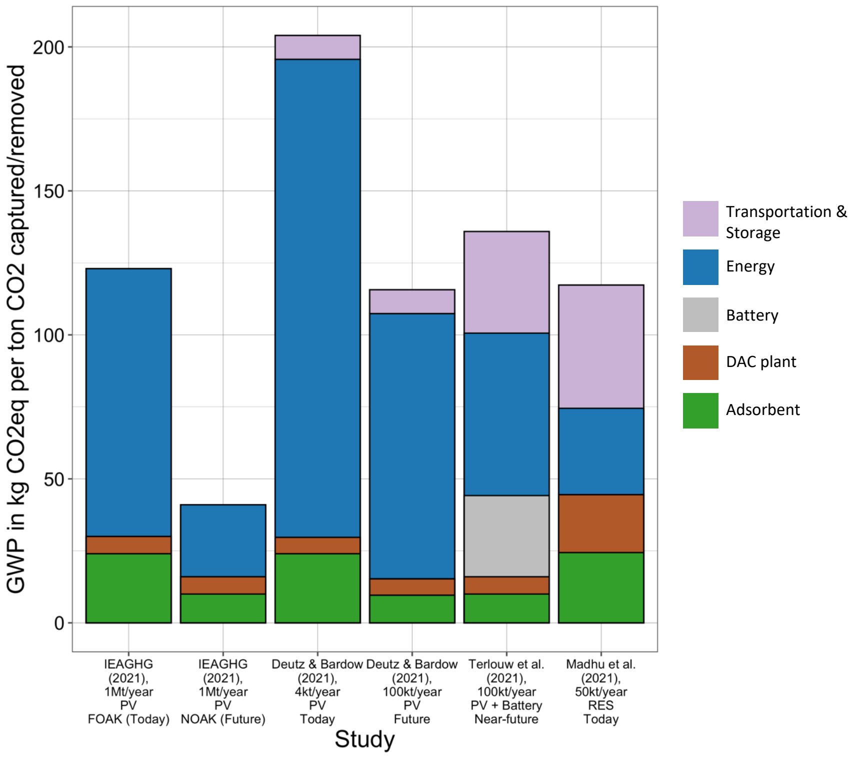

Supplementary Figure 1 Reported results for global warming potential associated with capture/removal of 1 ton CO_2 from the atmosphere with low-temperature, solid sorbent technology.

With Deutz & Bardow[1], Madhu et al.[2] and Terlouw et al.[3], three comprehensive LCAs of low-temperature DACS have recently been conducted. All share that adapted life cycle inventory (LCI) data from Climeworks´ DACS plant in Iceland was used. Since our interest lies in PV powered autonomous systems, presented results from reviewed studies focus on situations which come closest to this setting (see Supplementary Table 1). Results from the IEAGHG[4] report are also included, although authors of the report caution that no complete LCA was conducted.

Technology learning plays a crucial role for fast developing technologies such as DACSs. Moving from a reference 4 kt per year DAC to a future 100 kt per year DAC reduces specific heat requirements per ton CO_2 by 55% from 3,300 kWh_{heat} to 1,500 kWh_{heat} in Deutz & Bardow[1]. Specific electricity consumption comes down from 700 kWh to 500 kWh. These improvements, alongside lower adsorbent material consumption increase the CRE from 80% to 89% for a generic PV system in Germany as source of energy (see Supplementary Figure 1). Energy requirements obtained by Deutz & Bardow[1] have, with some adjustments, served as input for subsequent work of Terlouw et al.[3] and IEAGHG[4].

The IEAGHG compares a first of a kind (FOAK) DAC with a nth of a kind (NOAK), incorporating learning effects[4]. Energy requirement for the first 1 Mt per year plant was derived from Deutz & Bardow´s reference technology with a reduction of 10% due to a tenfold increase in capacity. Whether capacity increase by itself is associated with lower energy requirements is subject of debate. In an upscaling scenario from 50kt per year to 1Mt/year, Madhu et al.[2] reported reduced energy requirements only for HT liquid solvent systems but not LT solid sorbent ones due to the modular design of the latter. Reduction in energy related GHG emissions is more substantial in IEAGHG[4] than Deutz & Bardow[1]. This is explained by updates in the carbon intensity of PV electricity in IEAGHG[4] (from 50.9gCO_2 per kWh to 24.8gCO_2 per kWh from FOAK to NOAK) while Deutz & Bardow[1] use the same carbon footprint for PV in both scenarios.

Both Deutz & Bardow[1] and IEAGHG[4] did not include any impacts of intermittency in their PV setup. Consequently, presented results might overestimate the achievable CRE. Terlouw et al.[3] who included impacts of a LIB storage, report that the battery accounts for around 20% of GWP for carbon removal. However, only one battery configuration (221MWh) is looked at for PV-powered systems with a heat pump.

Recently, Qui et al.[5] evaluated effects of technology learning and updates of background inventory data on the environmental impacts of DAC (using a 100kt per year scenario from Deutz & Bardow[1] as a start). Results are interesting but not included in Supplementary Figure 1 since only grid-connected systems were assessed.

1.2 Cost assessments:

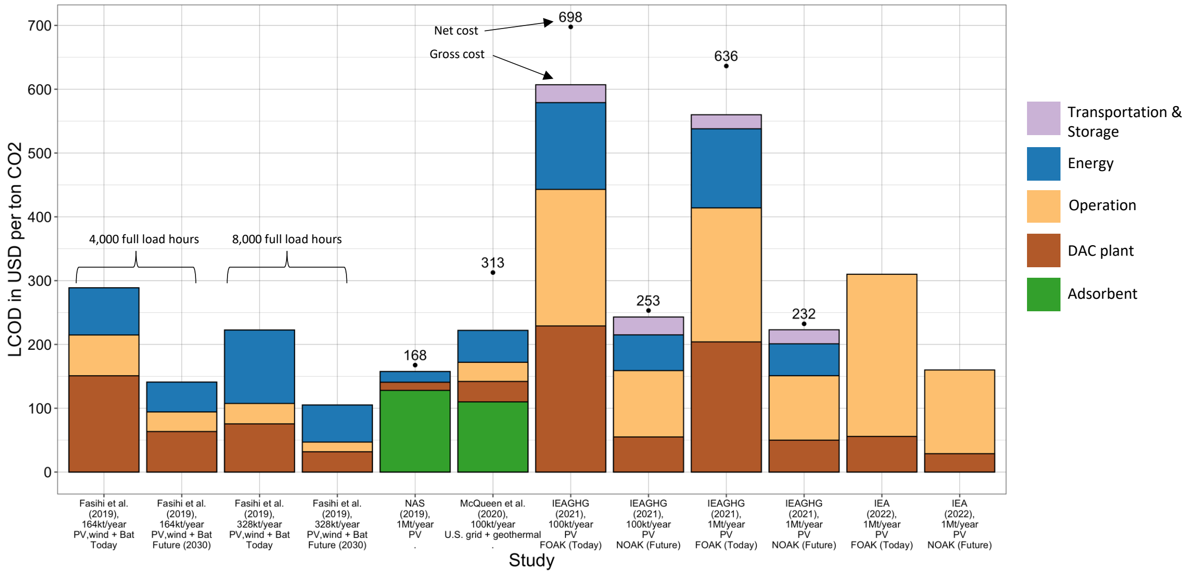

Supplementary Figure 2 Reported results for costs associated with capture/removal of 1 ton CO_2 from the atmosphere with low-temperature, solid sorbent technology.

In 2019, Fasihi et al.[6] provided a cost model for solid sorbent LT and HT solvent DAC based on an extensive literature review. A hypothetical LT DAC plant with a capacity 360,000 tCO_2 per year was assessed. Costs were calculated for 2020, 2030, 2040 and 2050, using learning-rates to account for decreases in energy consumption and capital expenditure of the DAC plant. Heat demand for the reference year of 2020 was estimated as 1,750 kWh_{heat} per tCO_2, which is comparable to Terlouw et al.[3] and the future scenario of Deutz & Bardow[1]. The reference for specific electricity consumption is, with 250 kWh in 2020, substantially lower than reported in LCAs. Also, Fasihi et al.[6] paid early interest to stand-alone designs of DACs, modelling an energy system using wind power and PV alongside a battery storage system. Their work acknowledged that intermitted RESs reduce the full load hours of the DAC plant. Two designs with 4,000 and 8,000 full load hours per year, corresponding to a used DAC capacity of ca. 165 and 330 $ktCO_2$ per year, were assessed. Lower utilization of the DAC with the 4,000 full load hours layout increased the contribution of plant annuity costs compared to the 8,000 full load hour design, see Supplementary Figure 2. On the other hand, specific energy costs were lower as less overcapacity must be provided to achieve 4,000 full load hours.

Reports from the IEA[7] and IEAGHG[4] for PV-powered systems have not accounted for reduced full load hours or additional costs associated with an energy storage medium. Learning effects between FOAK and NOAK were included by both IEA[7] and IEAGHG[4]. The IEAGHG[4] reports reference cost for a FOAK, powered by PV with a heat pump of $607 (100kt$CO_2$ per

year DAC) and \$560 (1MtCO_2 per year DAC). Through learning, costs come down by 60% to \$243 and \$232 for a NOAK plant. Combined with GWP estimates, IEAGHG[4] allows to estimate costs for net CO_2 removal (although only carbon capture efficiency, not carbon removal efficiency was calculated by IEAGHG in Supplementary Figure 1). IEA puts average costs for a 1MtCO_2 per year LT solid-sorbent FOAK and NOAK system at \$310 and \$160. Using average CRE calculated by LCAs, but not necessarily for the same system boundary, increases costs.

Cost estimates from the National Academy of Science (NAS)[8] use low energy requirements of ca. 1140 kWh in their middle scenario. Surprisingly, specific annuities associated with the capital expenditure of the DAC plant is less than \$20. Fasihi et al.[6], IEA[7] and IEAGHG[4] share that adsorbent costs are part of operation costs and cannot easily be divided into contribution of adsorbent material degradation and other operating costs, which would increase transparency and was done by McQueen et al.[9] The setup in McQueen et al.[9] for a 100ktCO_2 per year system is only in part comparable to a stand-alone system because electricity is used from the U.S. grid and heat as waste heat from a geothermal power plant.

Publication shown in Supplementary Table 1 and Supplementary Table 2 have served as benchmark for life cycle assessments and cost evaluations of solid-sorbent direct air carbon capture (and storage).

Supplementary Table 1 Publications of life cycle assessments included in benchmark review.

Study	Technology	Functional unit	Capacity [tCO_2 per year]	Locations	Heat demand (capture): kWh per tCO_2	Heat source	Electricity demand (capture) : kWh per tCO_2	Electricity source	Time	LCIA model	Impact categories	Capture or removal	Scaling
Deutz & Bardow[1]	LT solid sorbent (amine on silica) – Climeworks	FU1: 1 kg CO_2 captured, (FU2: 1 kg CO_2 removed)	S1: 4,000<br>S2: 100,000	Iceland, Germany for generic pv-system	S1: 3,300<br>S2: 1,500	Heat pump*	S1: 700<br>S2: 500	PV*	S1: today<br>S2: future	EF 2.0	16 EI from EF 2.0	Capture is focus, removal estimated	Technological learning from FOAK to NOAK (Changes of energy system only for grid in 2030 or 2050
Terlouw et al.[3]	LT solid sorbent - Climeworks	FU: "Gross removal of 1 ton CO_2 from the atmosphere via the use of a DAC plant combined with geological storage"	S1: 100,000	Jordan (L1), Greece (L2)*	S1: 1,500	Heat pump*	S1: 500	PV* (battery storage)	S1: near-future	EF 2.0, ReCiPe16	15 EI from EF 2.0, 1 EI from ReCiPe	Removal	Changes of energy system for grid-connected. Also update of background data but no results for stand-alone system. Upscaling from 4kt to 100kt with effect on less specific material for plant
Madhu et al.[2]	LT solid sorbent – Climeworks, (HT liquid – Carbon Engineering)	FU: "1 ton of CO_2 captured and stored"	S1: 50<br>S2: 1,000,000	Switzerland	S1: 720<br>S2: 720 (75% heat recovery rate, 2,890 without heat recovery)	Heat pump*	S1: 180<br>S2: 180	Low carbon* (ca 60% wind, 35% pv)	S1: today<br>S2: future	ReCiPe16	18 EI from ReCiPe	Removal	Same results for S1 and S2. Scaling has effect on liquid system but not solid sorbent. Results are same for S1,S2.
Qui et al.[5]	LT solid sorbent - Climeworks, (HT liquid - Carbon Engineering)	FU: „Capturing and sequestering one metric ton (1t) of atmospheric CO_2 by DACCS technologies"	S1: 100,000	USA*	1,500	Heat pump*	500	Grid electricity	2020 – 2100 (T1: 2020, T2: 2030, T3: 2040, T4: 2050)	ReCiPe16	8 EI from ReCiPe	Removal	Technology learning, changes in background energy system
IEAGHG[4]	LT solid sorbent, (HT liquid)		1,000,000		S1: 3,000<br>S2: 1,360 (both scaled from 100kt/year literature system with scaling factor of 1.1)	Heat pump*	S1: 640<br>S2: 440 (both scaled from 100kt/year literature system with scaling factor of 1.1)	PV*	S1: today<br>S2: 2050 (but could be earlier)	not specified	GHG	Capture	Technology learning FOAK, NOAK. Update of CO_2 intensity of pv electricity (from 50.9kg CO_2 per MWh in 2020 to 24.8 kg CO_2 per MWh in 2050<br>NOAK equivalent to 5-7 doublings of large-scale production capacity

*Other combinations have also been evaluated within the study.

Supplementary Table 2 Publications of cost calculation included in benchmark review.

Study	Technology	Reference unit	Capacity [tCO_2 per year]	Locations	Heat demand (capture): kWh per tCO_2	Heat source	Electricity demand (capture) : kWh per tCO_2	Electricity source	Time	Cost method	Capture or removal	Scaling
Fasihi et al.[6]	LT solid sorbent, (HT liquid sorbent)	1 ton CO_2 (gross) captured	360,000<br>C1: 4,000 full load hours<br>C2: 8,000 full load hours	Morocco	S1: 1750<br>S2: 1500<br>S3: 1286<br>S4: 1102	Heat pump*	S1: 250<br>S2: 225<br>S3: 203<br>S4: 182	Wind, PV*	S1: 2020,<br>S2: 2030,<br>S3: 2040,<br>S4: 2050	Levelized cost of CO_2 DAC (LCOD)	Capture	Learning applied to CAPEX, deployment of DAC based on literature, with DAC capacity of 15Gt per year in 2050.<br>Learning applied to energy demand. Cost decline in RES modelled.
NAS[8]	LT solid sorbent, (HT liquid sorbent)	1 Mt CO_2 captured and 1 Mt CO_2 net-captured (CCE calculated based on emission factors for energy)	1,000,000	USA	S2: 940<br>S4: 1333	Solar*	S2: 155<br>S4: 315	Solar*		"LCOD" (annualize d capex + opex)	Capture	No scaling?
McQueen et al.[9]	LT solid sorbent	1 ton CO_2 captured and 1 ton CO_2 net-captured	100,000	USA	1666	Waste heat geother mal*	416	U.S. Grid			Capture	Learning by doing (Capex scaled up from 10kt per year with $C_i = C_0 * (P_i/P_0)^\wedge(2/3)$
IEAGHG[4]	LT solid sorbent, (HT liquid)		C1: 100,000<br>C2: 1,000,000		S1: 3,000<br>S2: 1,360<br>(both scaled from 100kt/year literature system with scaling factor of 1.1)	Heat pump*	S1: 640<br>S2: 440<br>(both scaled from 100kt/year literature system with scaling factor of 1.1)	PV*	S1: today<br>S2: 2050 (but could be earlier)	LCOD	Removal (emission s only for capture)	Technology learning FOAK, NOAK. Update of CO_2 intensity of pv electricity (from 50.9kg CO_2 per MWh in 2020 to 24.8 kg CO_2 per MWh in 2050<br>NOAK equivalent to 5-7 doublings of large-scale production capacity
IEA[7]	LT solid sorbent, (HT liquid solvent)	1 ton CO_2 gross captured	1,000,000		1500 - 2110	Heat pump*	400 - 660	PV*	S1: today<br>S2: NOAK	LCOD	Removal	Technology learning from FOAK to NOAK. R&D, learning by doing and economies of scale as key components

*Other combinations have also been evaluated within the study, **conversion rate USD:€ of 1.1:1.

2. Input data for costs and environmental impacts

Input data for modelling the off-grid DACSs are presented in Supplementary Tables 3 and 4. Further, Supplementary Table 5 shows the scenario assumptions used for the evaluation of strategic improvements over the baseline reference.

Supplementary Table 3 Technological input data for off-grid DACS.

	Value	Unit	Sources
Design capacity	100,000	tCO_2 per year	
Specific electricity demand	0.6	MWh_{el} per tCO_2	[7-9]
Specific heat demand	3.33	MWh_{heat} per tCO_2	[1]
Specific electricity demand for heat with heat pump (COP of 2.6 for Nevada)	1.28	MWh_{el} per tCO_2	calculated
Specific electricity demand compression	0.1	MWh_{el} per tCO_2	[1-3]
Overall specific electricity demand	1.98	MWh_{el} per tCO_2	calculated
Distance to storage	0	km	
Adsorbent material consumption	7.5	kg per tCO_2	[1]
Lifetime of DAC plant	12.5	years	
Lifetime of PV	25	years	
Lifetime of Battery storage	12.5	years	
Lifetime of Heat Pump	25	years	

Supplementary Table 4 Cost and environmental input data for off-grid DACS.

	Value (year)	adjusted to 2023 with CEPCI (investments) or CPI (consumables)	Unit	Sources
Interest rate	10		%	
Costs				
Specific Capex DAC	1,130 (2020)	1,520	$*year per tCO_2	[4]
Specific Capex PV	850,000 (2021)	962,000	$ per MW	[10]
Specific Capex Battery	300,000 (2021)	339,000	$ per MWh	[11]
Specific Capex Heat pump	485,000 (2018)	645,000	$ per MW_{heat}	[12]
Specific cost adsorbent	24 (2020)	28	$ per kg	[4]
Specific cost storage	16 (2019)	21	$ per tCO_2	[13]
*Cradle-to-gate global warming potential***				
Specific GWP DAC	140		$kgCO_2eq$*year per tCO_2	[3]
Specific GWP PV	1,164,670		$kgCO_2eq$ per MW	[14,15]
Specific GWP Battery	121,061		$kgCO_2eq$ per MWh	[14,16]
Specific GWP Heat pump	156,371		$kgCO_2eq$ per MW_{heat}	[14]
Specific GWP adsorbent	2.3		$kgCO_2eq$ per kg	[2,14]
Specific GWP storage	0.31		$kgCO_2eq$ per tCO_2	[14]

** Impacts for climate change using EF 3.0

Supplementary Table 5 Strategic scenario analysis.

Scenario	Value	Unit
Cost oriented learning		
Spec. Capex DAC reduced by 50% (A1)	760	\$*year per tCO_2
Spec. cost of adsorbent reduced by 50% (A2)	14	\$ per kg
Technology oriented learning		
Specific energy demand reduced by 50% (B1)	0.99	kWh_{el} per tCO_2
Adsorbent material consumption reduced by 50% (B2)	3.75	kg per tCO_2

Supplementary Figure 3 shows the model graph of the DACS system as implemented in OpenLCA, in alignment with the system boundary shown in Figure 1 of the manuscript. Life cycle inventory data for the DAC plant was taken from ref.[3], which have provided a public proxy for a low-temperature, solid sorbent direct air capture system based on proprietary data from Climeworks which are not available publicly. While caution should be taken with the simplified infrastructure modelling for the DAC system, internal comparison by ref.[3] showed alignment between the simplified LCI data and the original data from Climeworks. The PEI adsorbent material was modelled as monomethylamine, following the approach of ref.[2]. Supplementary Figures 4 – 7 give detail on the LCI modelling of CO_2 storage, adsorbent material, DAC plant, and the energy system.

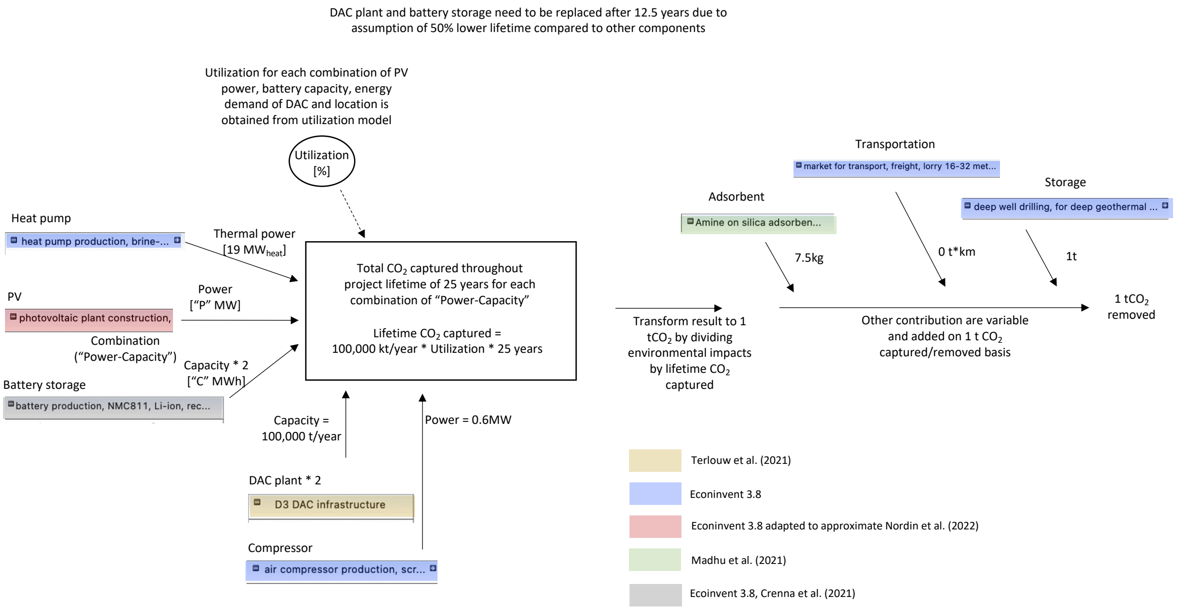

Supplementary Figure 3 Model graph LCA and data sources. Data from Terlouw et al.[3], Ecoinvent 3.8[14], Nordin et al.[15], Madhu et al.[2] and Crenna et al.[16]. Energy input data for reference scenario.

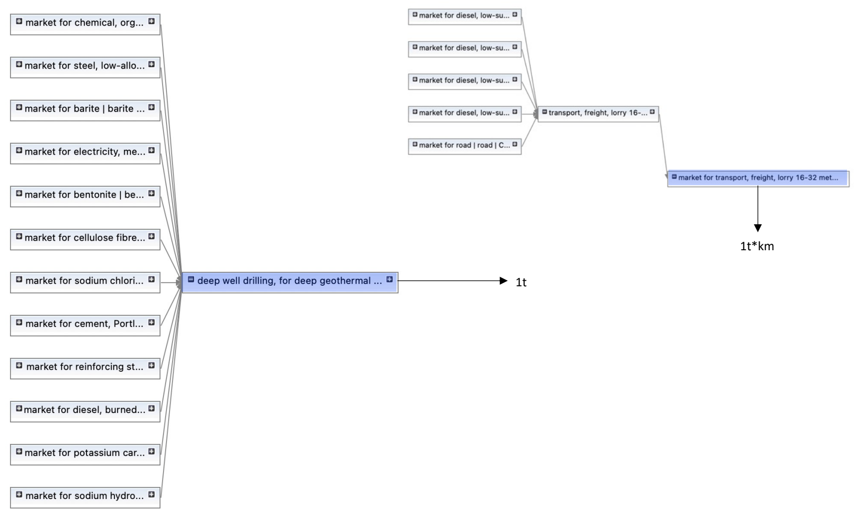

Supplementary Figure 4 Model graph transportation and storage, implemented in OpenLCA[17].

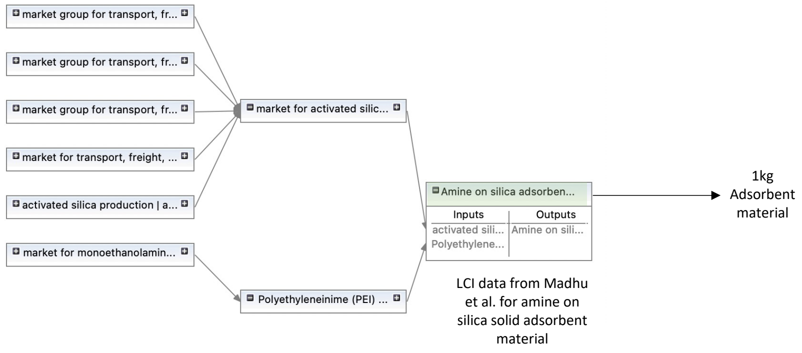

Supplementary Figure 5 Model graph adsorbent material, implemented in OpenLCA[17].

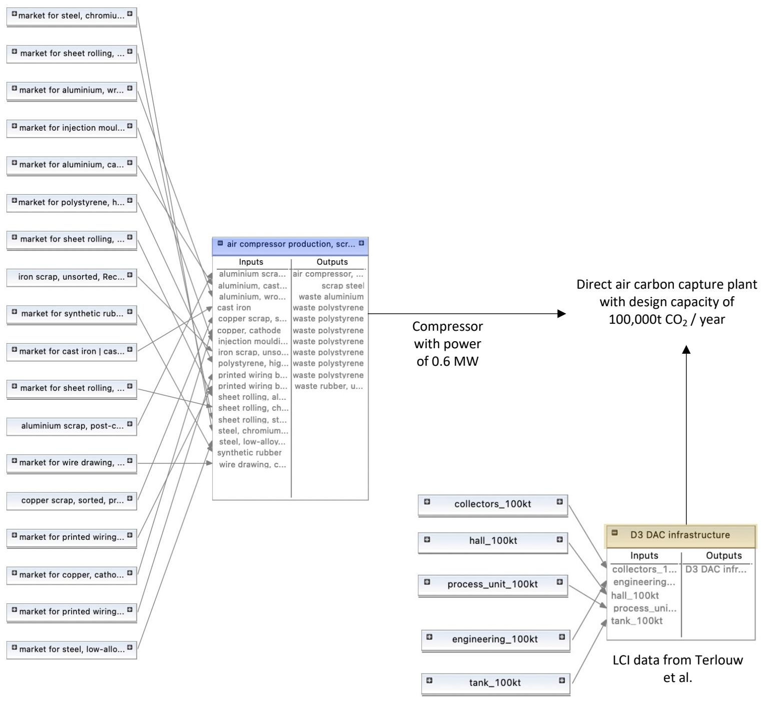

Supplementary Figure 6 Model graph direct air capture plant and compressor, implemented in OpenLCA[17].

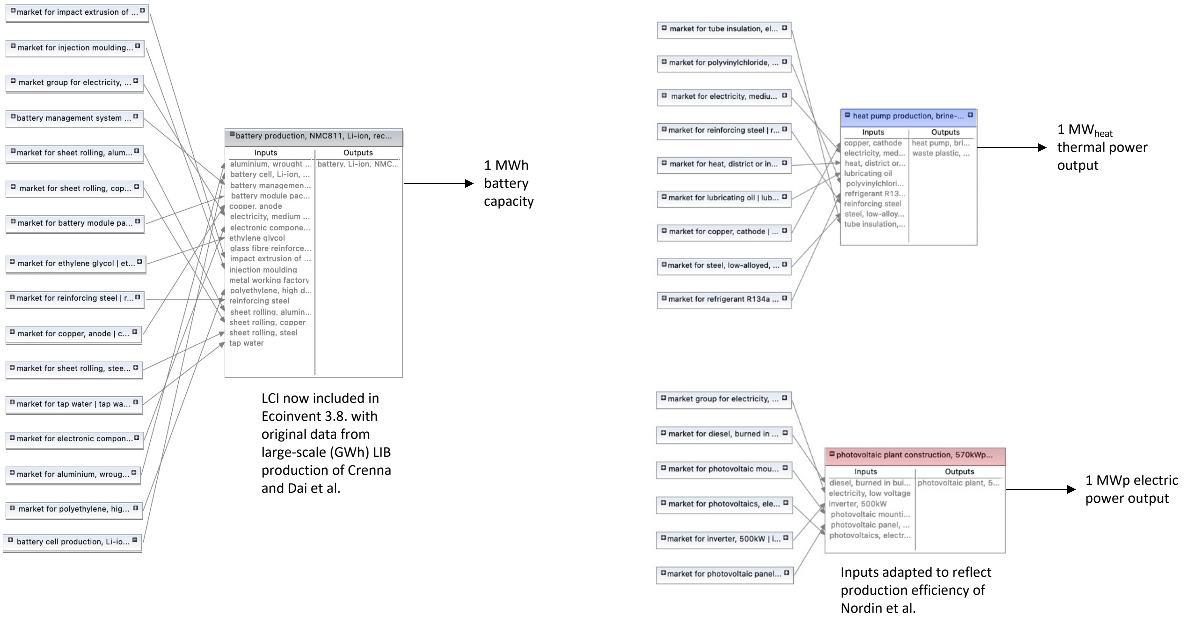

Supplementary Figure 7 Model graph photovoltaic system, battery storage and heat pump, implemented in OpenLCA[17].

Supplementary References

1 Deutz, S. & Bardow, A. Life-cycle assessment of an industrial direct air capture process based on temperature–vacuum swing adsorption. *Nat. Energy* **6**, 203-213, doi:10.1038/s41560-020-00771-9 (2021).

2 Madhu, K., Pauliuk, S., Dhathri, S. & Creutzig, F. Understanding environmental trade-offs and resource demand of direct air capture technologies through comparative life-cycle assessment. *Nat. Energy*, doi:10.1038/s41560-021-00922-6 (2021).

3 Terlouw, T., Treyer, K., Bauer, C. & Mazzotti, M. Life Cycle Assessment of Direct Air Carbon Capture and Storage with Low-Carbon Energy Sources. *Environ. Sci. Technol.* **55**, 11397-11411, doi:10.1021/acs.est.1c03263 (2021).

4 IEAGHG. Global Assessment of Direct Air Capture Costs. (2021).

5 Qiu, Y. *et al.* Environmental trade-offs of direct air capture technologies in climate change mitigation toward 2100. *Nature Communications* **13**, 3635, doi:10.1038/s41467-022-31146-1 (2022).

6 Fasihi, M., Efimova, O. & Breyer, C. Techno-economic assessment of CO 2 direct air capture plants. *J. Clean. Prod.* **224**, 957-980, doi:10.1016/j.jclepro.2019.03.086 (2019).

7 IEA. *Direct Air Capture 2022*. (IEA, 2022).

8 National Academies of Sciences Engineering & Medicine. *Negative Emissions Technologies and Reliable Sequestration: A Research Agenda*. (The National Academies Press, 2019).

9 McQueen, N. *et al.* Cost Analysis of Direct Air Capture and Sequestration Coupled to Low-Carbon Thermal Energy in the United States. *Environmental Science & Technology* **54**, 7542-7551, doi:10.1021/acs.est.0c00476 (2020).

10 IRENA. *Renewable Power Generation Costs in 2021.* (International Renewable Energy Agency, 2022).
11 Cole, W., Frazier, A. W. & Agustine, C. *Cost Projections for Utility-Scale Battery Storage: 2021 Update*. (National Renewable Energy Laboratory, 2021).
12 Pieper, H. *et al.* Allocation of investment costs for large-scale heat pumps supplying district heating. *Energy Procedia* **147**, 358-367, doi:10.1016/j.egypro.2018.07.104 (2018).
13 Smith, E. *et al.* The cost of CO2 transport and storage in global integrated assessment modeling. *Int. J. Greenh. Gas Control* **109**, 103367, doi:10.1016/j.ijggc.2021.103367 (2021).
14 Wernet, G. *et al.* The ecoinvent database version 3 (part I): overview and methodology. *Int. J. Life Cycle Assess.* **21**, 1218-1230, doi:10.1007/s11367-016-1087-8 (2016).
15 Nordin, A. H. M., Sulaiman, S. I., Shaari, S. & Mustapa, R. F. Energy and environmental impacts of a 37.57 MW dc ground-mounted large-scale photovoltaic system in Malaysia: A life-cycle approach. *J. Clean. Prod.* **335**, 130326, doi:10.1016/j.jclepro.2021.130326 (2022).
16 Crenna, E., Gauch, M., Widmer, R., Wäger, P. & Hischier, R. Towards more flexibility and transparency in life cycle inventories for Lithium-ion batteries. *Resources, Conservation and Recycling* **170**, 105619, doi:10.1016/j.resconrec.2021.105619 (2021).
17 Ciroth, A. ICT for environment in life cycle applications openLCA—A new open source software for life cycle assessment. *The international journal of life cycle assessment* **12**, 209-210 (2007).
